# Partial Inductance Model of Induction Machines for Fault Diagnosis

**DOI:** 10.3390/s18072340

**Published:** 2018-07-18

**Authors:** Manuel Pineda-Sanchez, Ruben Puche-Panadero, Javier Martinez-Roman, Angel Sapena-Bano, Martin Riera-Guasp, Juan Perez-Cruz

**Affiliations:** Institute for Energy Engineering, Universitat Politècnica de València, Cmno. de Vera s/n, 46022 Valencia, Spain; mpineda@die.upv.es (M.P.-S.); jmroman@die.upv.es (J.M.-R.); asapena@die.upv.es (A.S.-B.); mriera@die.upv.es (M.R.-G.); juperez@die.upv.es (J.P.-C.)

**Keywords:** partial inductance, fault diagnosis, condition monitoring, fast Fourier transform, induction machine

## Abstract

The development of advanced fault diagnostic systems for induction machines through the stator current requires accurate and fast models that can simulate the machine under faulty conditions, both in steady-state and in transient regime. These models are far more complex than the models used for healthy machines, because one of the effect of the faults is to change the winding configurations (broken bar faults, rotor asymmetries, and inter-turn short circuits) or the magnetic circuit (eccentricity and bearing faults). This produces a change of the self and mutual phase inductances, which induces in the stator currents the characteristic fault harmonics used to detect and to quantify the fault. The development of a machine model that can reflect these changes is a challenging task, which is addressed in this work with a novel approach, based on the concept of partial inductances. Instead of developing the machine model based on the phases’ coils, it is developed using the partial inductance of a single conductor, obtained through the magnetic vector potential, and combining the partial inductances of all the conductors with a fast Fourier transform for obtaining the phases’ inductances. The proposed method is validated using a commercial induction motor with forced broken bars.

## 1. Introduction

Induction machines (IMs) are key components of modern industrial processes, and their unexpected failures can generate unscheduled stops and high economic losses. To avoid this risk, on-line condition monitoring of IMs has become an integral part of industrial maintenance systems [1,2]. A great research effort has been devoted in the last decades to the development of fault diagnostic systems that can detect multiple machine faults in an early stage, giving a reliable information about the machine condition, and avoiding false alarms [3,4]. Different signals have been proposed to build fault diagnosis systems, such as vibrations [5], stator currents [6], thermal images [7], acoustic signals [8,9], etc. In particular, diagnostic algorithms that can detect the faults through their characteristic signature in the stator currents, known as motor current signal analysis (MCSA), have gain a great relevance [6,10], because they can be applied on-line using a simple hardware and software, just current clamps to measure the stator currents and the fast Fourier transform (FFT) to analyze them in the frequency domain [11,12]. Multiple types of faults can be detected by identifying the characteristic harmonics that they generate in the stator current, with frequencies that have been established theoretically.

For example, the characteristic frequencies of the fault harmonic components are, for bar breakages in squirrel cage induction machines [12,13,14] and rotor asymmetries in wound-rotor induction machines $f_{bb}$ [15]

(1) $$f_{bb}=\left(1+2ks\right)f_{1}\;k=\pm 1,\pm 2,\pm 3\ldots$$

for mixed eccentricity faults $f_{ecc}$ [16,17,18]

(2) $$f_{ecc}=f_{1}\left(1+k\frac{1-s}{p}\right)\;k=\pm 1,\pm 2,\pm 3\ldots$$

for inter-turn short-circuits in the stator winding $f_{sch}$ [19,20,21,22]

(3) $$f_{sch}=f_{1}\left(m\frac{1-s}{p}\pm k\right)\;m=0,1,2,\ldots ,k=0,1,3,\ldots$$

and for mechanical looseness $f_{loose}$ [23]

(4) $$f_{loose}=f_{1}\left(1+k\frac{1-s}{p\cdot n}\right)\;k=1,2,\ldots ,n=2,3,\ldots$$

where *s* is the slip, $f_{1}$ is the frequency of the power supply and *p* is the pole pairs number. Other types of faults, such as bearing faults [24,25,26], or gearbox faults [27] generate components in the stator current with frequency expressions similar to Equations (1)–(4). These equations are valid both in steady-state regime and in transient regime, but in this case the frequencies given by Equations (1)–(4) change along the time [15], and advanced time–frequency transforms must be used instead of the FFT [25,28].

Commonly, fault diagnostic systems that are based on the analysis of the current signal, such as MCSA, try to measure the amplitude of the harmonic components with frequencies given by Equations (1)–(4) in the spectrum of the stator current. These values are then compared with predefined threshold alarms, in data based diagnostic systems [5], or with the output of machine models, in model based diagnostic systems [5,20,29,30,31]. In both cases, accurate machine models are needed, for fixing the values of the threshold alarms, for running the model-based fault diagnostic system [32], or for developing new diagnostic algorithms [33]. The most accurate machine models are built using the finite elements analysis (FEA) [34], because it can take into account the actual geometry of the machine, including the asymmetries induced by the fault (eccentric machines), the characteristic of the magnetic materials, which depend on their operating point (saturation, hysteresis), and the configuration of the windings, which can be highly asymmetrical in case of rotor asymmetries or inter-turn short-circuits faults [35]. However, FEA models demand a great amount of computational resources and computing time [17]. This is a serious drawback for using FEA models on embedded on-line diagnostic hardware [24], such as DSPs or FPGAs [36], which have limited computing resources, or in systems which require a fast response, such as real-time model-based diagnostic systems [37]. A growing trend is the use of the IM model in hardware-in-the-loop (HIL) systems, which are used for developing and testing real-time diagnostic hardware, [38], or for training advanced artificial intelligence (AI) based diagnostic systems, such as the set membership identification (SMI) approach [14], neural networks [39,40,41], support vector machines [42] or fuzzy inference systems [43].

To alleviate these drawbacks, several solutions have been proposed in the technical literature, such the use of coupled finite elements and circuit equations [44], improved conformal mappings [45], or field reconstruction methods (FRM), based on linear superposition of FEA solutions [46]. An alternative approach to FEA models are fast and simpler analytical models of the induction machine [47], such as the magnetic equivalent circuit (MEC) model [36,48], which represents the induction machine as a mesh of reluctance elements, or the permeance network model (PNM) [49]. Recent works also suggest the use of generalized two-phase model of an induction machine of fifth order [50], space phasor models [27], complex-vector models [51], or equivalent circuit models [52].

Among the analytical models, the multiple coupled circuit model (MCCM) [53,54], also called the winding function approach [55], has been extensively used for modeling the IM in faulty conditions [56,57,58]. As Ojaghi and Faiz [55] pointed out, it is the most detailed and complete model used to analyze the performance of cage IMs, especially under various faults. It relies on the time-varying self and mutual inductances of the machine phases and rotor-loops, and their derivatives, which are calculated by integral expressions representing the placement of the winding turns along the air-gap periphery. However, these integral expressions must be computed for every rotor position and phase winding layout, which can be altered due to the asymmetries generated by the machine’s fault, resulting in the need of heavy computing resources to build the machine inductances matrices under faulty conditions. As it is stated in [54], this task typically consumes a high amount of time, so that only discrete curves of inductance versus rotor position are calculated and linear interpolation is applied at intermediate rotor positions. To solve this problem, in this paper, a novel, fast approach is proposed for obtaining the matrices of self and mutual inductances, making use of the concept of partial inductance of a single conductor [59,60]. Instead of solving the integral expression for every different winding layout, the partial inductance of a single conductor [61] is computed, using the magnetic vector potential (MVP), and the total self and mutual inductances of the windings are obtained by simply combining the partial inductances of their conductors. This combination is made in a very fast way using the fast Fourier transform. The partial inductance approach has been used in the partial element equivalent circuit (PEEC) method [62] for the electromagnetic modeling of power electronic modules [63], antennas [64], high voltage transmission lines [65] or printed circuit boards (PCBs) [66], but it is the first time, to the authors’ best knowledge, that it is proposed for obtaining the phase inductances of IMs. Compared with the WFA, this novel method is characterized by the following main points:

1. The conductor, instead of the coil, is used as the basic winding unit, which simplifies the modeling of arbitrarily complex winding layouts.
2. The partial inductance of a single conductor, computed using the MVP, is taken as the characteristic function of the machine’s windings, instead of the winding functions based on the magnetomotive force (MMF) of the coils.
3. A fast circular convolution, computed through the FFT, is used to obtain the mutual and self inductances of the phases, instead of solving the integral expressions of the winding functions for every rotor position.

The structure of this paper is as follows. In Section 2, the electro-mechanical equations used for modeling the IM are briefly presented. In Section 3, the partial inductance of a single conductor is obtained through its MVP, and in Section 4 the FFT is used for assembling the matrices of self and mutual inductances of the machine, combining the partial inductances of the phases’s conductors. Section 5 presents the experimental validation of the proposed approach, using a commercial IM with broken bar faults. Finally, Section 6 presents the conclusions of this work.

## 2. Electro-Mechanical System of Equations of an Induction Machine

The following system of equations can be written for an induction machine with *m* stator and *n* rotor phases with arbitrary layout, that is, even under fault conditions, [67]

(5) $$\left[U_{s}\right]=\left[R_{s}\right]\left[I_{s}\right]+d\left[\Psi_{s}\right]/dt$$

(6) $$\left[0\right]=\left[R_{r}\right]\left[I_{r}\right]+d\left[\Psi_{r}\right]/dt$$

(7) $$\left[\Psi_{s}\right]=\left[L_{ss}\right]\left[I_{s}\right]+\left[L_{sr}\right]\left[I_{r}\right]$$

(8) $$\left[\Psi_{r}\right]={\left[L_{sr}\right]}^{T}\left[I_{s}\right]+\left[L_{rr}\right]\left[I_{r}\right]$$

(9) $$\left[U_{s}\right]={\left[u_{s1},u_{s2},\ldots ,u_{sm}\right]}^{T}$$

(10) $$\left[I_{s}\right]={\left[i_{s1},i_{s2},\ldots ,i_{sm}\right]}^{T},\;\left[I_{r}\right]={\left[i_{r1},i_{r2},\ldots ,i_{rn}\right]}^{T}$$

where [U] is the phase voltages matrix, [I] is the phase currents matrix, [R] is the resistances matrix, [Ψ] is the flux linkages matrix and [L] is the inductances matrix. Subscripts *s* and *r* are used for the stator and for the rotor, respectively. The mechanical equations are

(11) $$T_{e}={\left[I_{s}\right]}^{T}\frac{\partial [L_{sr}]}{\partial \varphi }\left[I_{r}\right]$$

(12) $$T_{e}-T_{L}=J\frac{d\Omega }{dt}=J\frac{d^{2}\theta }{dt^{2}}$$

where $T_{e}$ is the electromechanical torque of the machine, $T_{L}$ is the load torque, *J* is the total system inertia (rotor plus load), Ω is the mechanical speed, θ is the angular position of the rotor, and φ is the angular coordinate. Two additional constraints, namely the sum of all the stator currents equals 0, and the sum of all the rotor currents equals 0, must be added to this set of equations.

**Figure 1:**
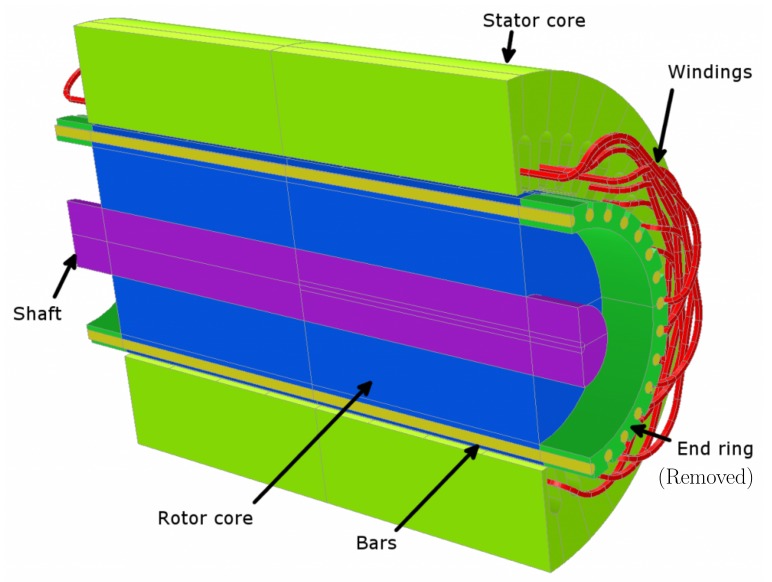
Stator windings and rotor bars of a three-phase squirrel cage induction motor.

To solve the system of Equations (5)–(12), the inductance matrices and their derivatives must be calculated very accurately, in order to be able to reproduce the fault harmonics given by Equations (1)–(4). The inductances in Equations (7), (8) and (11) correspond to the self and mutual inductances of the machine’s phases, and of the rotor loops in the case of squirrel cage machines (two consecutive bars and the sections of the end-rings between them). The inductances of these windings, such as the ones represented in Figure 1, can be computed summing up the coil inductances for all the phases’ coils, as in WFA. However, this approach must deal with the wide variety of coil types that can be used for building a phase winding. Besides, these inductances may change with the rotor position, as in the case of rotor-stator mutual inductances, and also in the case of winding faults, such as inter-turn short-circuits, or bar breakages. This approach results in complex and lengthy computations that typically consume a high amount of time [54]. The use of the partial inductance concept can greatly simplify this process, because the partial inductance of the conductor, instead of the coil inductance, is chosen as the building block for the computation of the inductance matrices, which is performed using a very simple and efficient FFT-based procedure, even in the case of complex or faulty winding layouts.

In this work, the following assumptions will be made for calculating the phase inductances, common to many analytical IM models presented in the technical literature:

1. There is negligible saturation.
2. There is a uniform air-gap.
3. The eddy currents are neglected. The windings are considered to be made of filamentary conductors placed on the external rotor surface or on the internal stator surface.
4. The end-coil leakage inductances need to be pre-calculated, and are treated as constants in Equations (7), (8) and (11), using explicit expressions that can be found in [52,68].

Some of these assumptions can be removed by using a modified inverse air-gap function which takes into account the saturation [55], the rotor eccentricity [30], or the slotting effects [69]. Nevertheless, as the focus of this paper is the introduction of the partial inductance model of the IM, a constant air-gap has been used to present the proposed approach, applied to the simulation and experimental test of an IM with a rotor asymmetry (broken bars fault), the same fault analyzed in [11,12,14,15,56], among many others.

## 3. Phase Inductance as a Sum of the Conductors’ Partial Inductances

**Figure 2:**
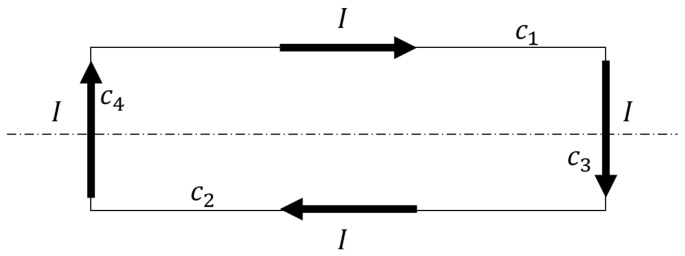
Rectangular current loop representing a winding coil.

Let us consider a single coil of the machine windings, represented as a rectangular current loop in Figure 2, where conductors $c_{1}$ and $c_{2}$ are parallel to the machine’s axis, and conductors $c_{3}$ and $c_{4}$ belong to the end windings.

The self inductance of this single coil, under the assumptions given in Section 2, is given by Equation

(13) $$L_{coil}=\frac{\Psi }{I}$$

where Ψ and *I* are the flux linked by the coil and its current, respectively, assuming that *I* is the only current in the machine. The same equation holds for the mutual inductance between two coils, just using the current of the second coil in Equation (13). However, the coil inductance given by Equation (13) is a global coil characteristic which can’t be assigned to any segment of the loop represented in Figure 2. On the contrary, a partial inductance can be assigned individually to each of the conductors that forms the coil loop. In effect, Equation (13) can be expressed as Equation

(14) $$L_{coil}=\frac{\int_{S}B\cdot dS}{I}$$

where B is the magnetic flux density through the loop area S. Using B=∇×A, where A is the magnetic vector potential, and applying Stokes’ theorem, gives Equation

(15) $$L_{coil}=\frac{\int_{S}\nabla \times A\cdot dS}{I}=\frac{\oint_{C}A\cdot dl}{I}$$

where *C* is the contour of the current loop. However, the contour integral in Equation (15) can be broken into the four segments of the loop of Figure 2 as

(16) $$\begin{array}{c} L_{coil}=\frac{\int_{C_{1}}A\cdot dl}{I}+\frac{\int_{C_{2}}A\cdot dl}{I}+\frac{\int_{C_{3}}A\cdot dl}{I}+\frac{\int_{C_{4}}A\cdot dl}{I}\\ =L_{coil_{1}}+L_{coil_{2}}+L_{coil_{3}}+L_{coil_{4}} \end{array}$$

where $C_{i}$ is the contour of each segment of the loop. The net inductance of each segment $C_{i}$, $L_{i}$ in Equation (16), represents the contribution of the segment $C_{i}$ to the total inductance of the current loop.

Using the concept of partial inductance, the inductance of each segment *i* of the coil in Equation (16) $L_{coil_{i}}$ (i=1…4) can be expressed as the sum of the partial inductances of segment *i* in Equation

(17) $$L_{coil_{i}}=\frac{\int_{C_{i}}A\cdot dl}{I}=\sum_{j=1}^{4}Lp_{ij}$$

where the partial inductance $Lp_{ij}$ is defined [59,60] as the ratio between the magnetic flux crossing the rectangular surface between the segment *i* and infinity, and the current $I_{j}$ that produces that flux (which, in this case, is the same for all the loop conductors). That is,

(18) $$Lp_{ij}=\frac{\int_{C_{i}}A_{\mathrm{ij}}\cdot dl}{I_{j}}$$

Using Equations (16) and (17), the total inductance of the rectangular loop in Equation (13) can be expressed as the sum of all the partial inductances of its segments, as

(19) $$L_{coil}=\sum_{i=1}^{4}\sum_{j=1}^{4}Lp_{ij}$$

As stated in the previous section, the end-coil leakage inductance is considered in this work as a constant value, computed a priori, so Equation (19) must be applied only to the axial conductors of the coil, $C_{1}$ and $C_{2}$ in Figure 2. With this assumption, the sums in Equation (19) must be extended only to these two conductors, as

(20) $$L_{coil}=\sum_{i=1}^{2}\sum_{j=1}^{2}Lp_{ij}+L_{end\_coil}$$

In a similar way, the self-inductance of a phase *A* with $N_{A}$ conductors can be computed extending Equation (20) to account for all the phase’s conductors, as

(21) $$L_{phase_{A}}=\sum_{i=1}^{N_{A}}\sum_{j=1}^{N_{A}}Lp_{ij}+L_{end\_phase}$$

where $L_{end\_phase}$ represents the total end-winding leakage inductance of the phase.

The expression for the mutual inductance between phase *A* and other phase *B*, with $N_{B}$ conductors, is analogous to Equation (21),

(22) $$L_{AB}=\sum_{i=1}^{N_{A}}\sum_{j=1}^{N_{B}}Lp_{ij}$$

with the additional assumption of neglecting the mutual inductance between the end-coils of both phases.

Comparing Equations (21) and (22), it is clear that $L_{phase_{A}}$ in Equation (21) can be obtained using Equation (22) as

(23) $$L_{phase_{A}}=L_{AA}+L_{end\_phase}$$

so this work will focus in the effective computation of Equation (22).

The practical implementation of the proposed method seems to be cumbersome. First, a partial differential equation (PDE) must be solved for every conductor to obtain the MVP in Equation (18). Second, two double summations must be made in Equations (21) and (22), for all the stator and rotor phases. This process must be repeated for every position of the rotor, for obtaining the mutual inductance between a rotor phase and a stator phase. Nevertheless, in the next sections, an analytical expression for Equation (18) is derived, and a simple procedure is given for obtaining Equation (22), for any possible displacement between phases *A* and *B*, using a single FFT.

### 3.1. Partial Inductance between Axial Conductors

As only the partial inductances between the axial conductors of the phases are considered in Equations (21) and (22), only the axial component of the MVP must be taken into account. Neglecting end effects, a central cross section, perpendicular to the machine axis, is analyzed, thus using a 2D approximation for computing the MVP. Under these conditions, the MVP has a single, axial component, $A_{z}$. If the length of the axial conductors is equal to the effective length of the magnetic core of the machine $l_{maq}$, the value of $Lp_{ij}$ in Equation (18) is simply

(24) $$Lp_{ij}=\frac{Az_{ij}\cdot l_{maq}}{I_{j}}$$

In an induction machine, the boundary condition at the infinity in Equation (18) can be replaced with a zero Dirichlet boundary condition at the external surface of the stator, where the MVP is assumed to be zero (no flux crossing the external surface of the stator). With this boundary condition, $Az_{ij}$ in Equation (18) is just the MVP at the conductor’s position, which is equal to the yoke flux per unit length at this position.

The method used in this work for obtaining the partial inductance $Lp_{ij}$ in Equation (24) between two axial conductors *i* and *j* consists in feeding conductor *j* with a unit current, being this the only current in the machine, and computing the MVP at the position of conductor *i*, so that Equation (24) becomes

(25) $$Lp_{ij}=l_{maq}\cdot Az_{ij}$$

**Figure 3:**
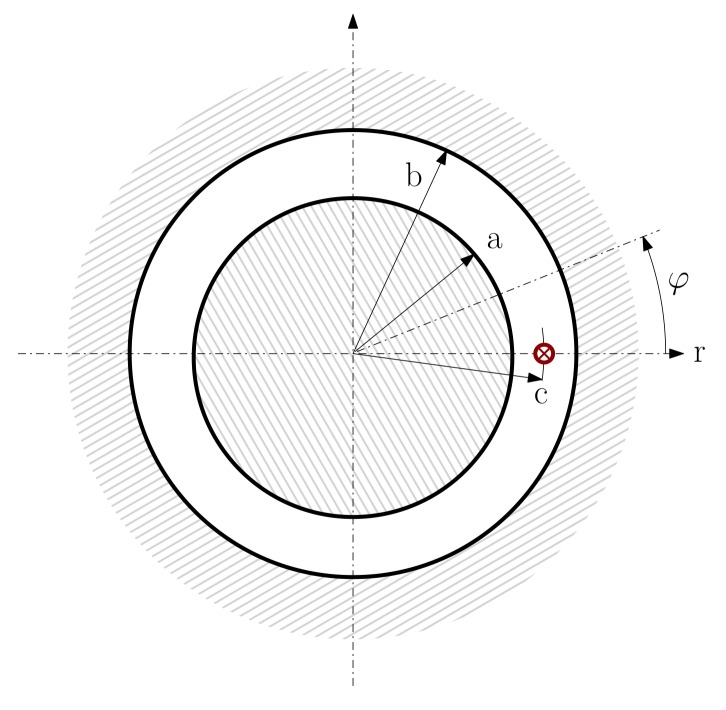
Linear current in an air-gap with concentric circular cylindrical boundaries.

For applying the proposed method, it is necessary to obtain the MVP generated by a single conductor, fed with a unit current. The MVP generated by a linear current in an air-gap with concentric circular cylindrical boundaries, as shown in Figure 3, must satisfy the Poisson’s equation

(26) $$\frac{\partial^{2}Az\left(r,\varphi \right)}{\partial r^{2}}+\frac{1}{r}\frac{\partial Az(r,\varphi )}{\partial r}+\frac{1}{r^{2}}\frac{\partial^{2}Az\left(r,\varphi \right)}{\partial \varphi^{2}}=-\mu_{0}J\left(r,\varphi \right)$$

Equation (26) can be solved using the method of separation of variables, as in [70,71]. Placing the conductor at the origin of the angular coordinate (φ=0 in Figure 3), and assuming that the iron of the cylindrical boundaries is infinitely permeable, the solution of Equation (26) for the conductor shown in Figure 3 is

(27) $$Az_{0}\left(r,\varphi \right)=\frac{\mu_{0}J}{\pi }\times \left\{\begin{array}{cc} \sum_{n=1}^{\infty }\frac{1}{n\cdot c^{n}}\frac{c^{2n}+b^{2n}}{b^{2n}-a^{2n}}\frac{r^{2n}+a^{2n}}{r^{n}}\cos\left(n\varphi \right)-\ln\left(c\right) & r\leq c\\ \sum_{n=1}^{\infty }\frac{1}{n\cdot c^{n}}\frac{c^{2n}+a^{2n}}{b^{2n}-a^{2n}}\frac{r^{2n}+b^{2n}}{r^{n}}\cos\left(n\varphi \right)-\ln\left(r\right) & r>c \end{array}\right.$$

**Figure 4:**
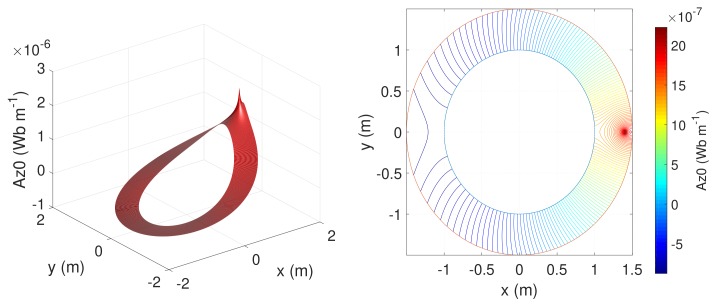
(**Left**) MVP that produces in the air-gap an axial conductor located at φ=0 and r=c when fed with a 1A current; and (**right**) induction lines in the air-gap.

The constant, cyclic part of Equation (27) (ln(c),ln(r)) can be discarded because, in any induction machine, the net sum of the linear currents through a transversal section is always zero. Figure 4 plots the harmonic part of Equation (27) for an example machine with a=1, c=1.4 and b=1.5.

**Figure 5:**
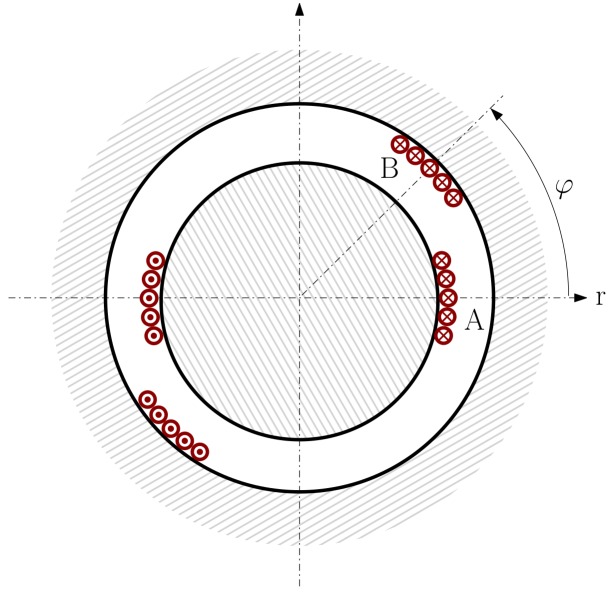
Location of the winding conductors in the air-gap on the outer surface of the rotor (phase **A**) and on the inner surface of the stator (phase **B**), for two phases separated an angular distance φ.

If the conductors are assumed to lie on the outer rotor surface or in the inner stator surface (Figure 5), then the linear currents are restricted to c=a or c=b in Figure 5. Besides, for obtaining the partial inductances of the conductors, the MVP must be calculated only on these same surfaces, that is, r=a or r=b in Figure 5. These four possibilities (c=a,c=b,r=a,r=b) reduce Equation (27) to only two different expressions: the MVP generated by an axial conductor on the same surface where it lies, or on the opposite one:

(28) $$Az_{0}\left(\varphi \right)=\frac{\mu_{0}J}{\pi }\times \left\{\begin{array}{cc} \sum_{n=1}^{\infty }\frac{1}{n}\frac{b^{2n}+a^{2n}}{b^{2n}-a^{2n}}\cos\left(n\varphi \right) & \mathrm{same}\;\mathrm{surface}\\ \sum_{n=1}^{\infty }\frac{1}{n}\frac{2a^{n}b^{n}}{b^{2n}-a^{2n}}\cos\left(n\varphi \right) & \mathrm{opposite}\;\mathrm{surface} \end{array}\right.$$

The partial inductance of Equation (25) between two axial conductors *i* and *j* can now be computed using the values given by Equation (28). If conductor *i* is placed at the origin (φ=0 in Figure 3), and conductor *j* is placed at an arbitrary angular position φ, then the partial inductance between these two axial conductors is

(29) $$Lp_{0}\left(\varphi \right)=\frac{\mu_{0}\cdot l_{maq}}{\pi }\times \left\{\begin{array}{cc} \sum_{n=1}^{\infty }\frac{1}{n}\frac{b^{2n}+a^{2n}}{b^{2n}-a^{2n}}\cos\left(n\varphi \right) & \mathrm{same}\;\mathrm{surface}\\ \sum_{n=1}^{\infty }\frac{1}{n}\frac{2a^{n}b^{n}}{b^{2n}-a^{2n}}\cos\left(n\varphi \right) & \mathrm{opposite}\;\mathrm{surface} \end{array}\right.$$

If now conductor *i* is assumed to be at an angular position φ=α, instead of φ=0, then the partial inductance with conductor *j* will be simply

(30) $$Lp_{\alpha }\left(\varphi \right)=Lp_{0}\left(\varphi -\alpha \right)$$

The derivative of the partial inductance with respect to the angle, which is used for computing the torque in Equation (11), can be obtained directly from Equation (29) as

(31) $$\frac{\partial Lp_{0}\left(\varphi \right)}{\partial \varphi }=-\frac{\mu_{0}\cdot l_{maq}}{\pi }\times \sum_{n=1}^{\infty }\frac{2a^{n}b^{n}}{b^{2n}-a^{2n}}\sin\left(n\varphi \right)$$

In Equation (31), only the derivative of the mutual inductance between conductors in opposite surfaces is needed, due to the uniformity of the air-gap.

### 3.2. Discretization of the Expression of the Partial Inductance between Axial Conductors

Equation (29) must be discretized in order to be used in an induction machine’s model. Therefore, the air-gap circumference is divided into *N* equal-length intervals, with an angular width equal to Δφ=2π/N. In this case, the maximum number of harmonics of the infinite series in Equation (29) that are needed to represent the partial inductance is, by Shannon’s theorem, N/2. Thus, the sequence that gives the partial inductance between a conductor placed at the origin, and other conductor *j* placed at an angular position j·Δφ, with j=0…N−1, is

(32) $$Lp_{0}\left[j\right]=\frac{\mu_{0}\cdot l_{maq}}{\pi }\times \left\{\begin{array}{cc} \sum_{n=1}^{n=N/2-1}\frac{1}{n}\frac{b^{2n}+a^{2n}}{b^{2n}-a^{2n}}\cos\left(n\cdot j\Delta \varphi \right) & \mathrm{same}\;\mathrm{surface}\\ \sum_{n=1}^{n=N/2-1}\frac{1}{n}\frac{2a^{n}b^{n}}{b^{2n}-a^{2n}}\cos\left(n\cdot j\Delta \varphi \right) & \mathrm{opposite}\;\mathrm{surface} \end{array}\right.$$

Analogously to Equation (30), if conductor *i* is not placed at the origin, but at an angular position i·Δφ, then Equation (32) becomes

(33) $$Lp_{i}\left[j\right]=Lp_{0}\left[{\left(\left(j-i\right)\right)}_{N}\right]$$

where ${\left(\left(j-i\right)\right)}_{N}=\left|j-i\right|\;\mathrm{mod}\;N$ is understood as a modulo *N* operation. In this work, to simplify the mathematical expressions, all the matrix indexes are considered as modulo *N*, without using the full notation of Equation (33). Following this convention, Equation (33) is expressed as

(34) $$Lp_{i}\left[j\right]=Lp_{0}\left[j-i\right]$$

The derivative with respect to the angular coordinate of the partial inductance between two conductors placed on opposite surfaces, given by Equation (31), can be also discretized with the same procedure, giving a sequence defined by

(35) $$dLp_{0}\left[j\right]=-\frac{\mu_{0}\cdot l_{maq}}{\pi }\times \sum_{n=1}^{n=N/2-1}\frac{2a^{n}b^{n}}{b^{2n}-a^{2n}}\sin\left(n\cdot j\Delta \varphi \right)$$

## 4. Computation of the Mutual Inductances between Two Phases Based on the Partial Inductance between Two Axial Conductors

### 4.1. Vectors Containing the Distribution of Conductors of Phases A and B and the Partial Inductances between Two Axial Conductors

The process for computing the mutual inductance between two phases *A* and *B*, such as those represented in Figure 5, is based on the substitution in Equation (22) of Equation (33). To perform this process, using discrete arithmetic, three column vectors of *N* elements are needed.

- The vector ${\vec{L}}_{p0}$, whose element *j*, $L_{p0}\left[j\right]$, contains the partial inductance between a conductor placed at the origin and other conductor placed at an angular position j·Δφ, using the corresponding Equation (32) (depending if both conductors are in the same surface or in opposite surfaces).
  (36) $${\vec{L}}_{p0}=\left\{L_{p0}\left[0\right],L_{p0}\left[1\right],\ldots ,L_{p0}\left[N-1\right]\right\}$$
- The vector ${\vec{dL}}_{p0}$, whose element *j*, $dL_{p0}\left[j\right]$, contains the derivative with respect to the angular coordinate of the partial inductance between a conductor placed at the origin and other conductor placed at an angular position j·Δφ, using the corresponding Equation (35).
  (37) $$\frac{\partial {\vec{L}}_{p0}}{\partial \varphi }={\vec{dL}}_{p0}=\left\{dL_{p0}\left[0\right],dL_{p0}\left[1\right],\ldots ,dL_{p0}\left[N-1\right]\right\}$$
- The vector ${\vec{Z}}_{A0}$, whose element *j*, $n_{A0}\left[j\right]$, contains the number of conductors of phase *A* located in the angular interval of length Δφ centered at position j·Δφ. The direction of the currents at each position is represented using positive and negative values, depending on the current direction
  (38) $${\vec{Z}}_{A0}=\left\{Z_{A0}\left[0\right],Z_{A0}\left[1\right],\ldots ,Z_{A0}\left[N-1\right]\right\}$$
  In the definition of ${\vec{Z}}_{A0}$, it is assumed that the axis of the distribution of conductors of the phase coincides with the origin of coordinates φ=0. If the phase is shifted by an angle $\varphi_{A}=k_{A}\cdot \Delta \varphi$, then all the elements of ${\vec{Z}}_{A0}$ are shifted by $k_{A}$, that is
  (39) $$Z_{Ak_{A}}\left[j\right]=Z_{A0}\left[j-k_{A}\right]$$
- The vector ${\vec{Z}}_{B0}$, which contains the distribution in the airgap of the conductors of phase *B*, which is defined in the same way as vector ${\vec{Z}}_{A0}$
  (40) $${\vec{Z}}_{B0}=\left\{Z_{B0}\left[0\right],Z_{B0}\left[1\right],\ldots ,Z_{B0}\left[N-1\right]\right\}$$
  In a similar way to the definition of ${\vec{Z}}_{A0}$, it is assumed that the axis of the distribution of conductors ${\vec{Z}}_{B0}$ coincides with the origin of coordinates φ=0. If the phase is shifted by an angle $\varphi_{B}=k_{B}\cdot \Delta \varphi$, then all the elements of ${\vec{Z}}_{B0}$ are shifted by $k_{B}$, that is
  (41) $$Z_{Bk_{B}}\left[j\right]=Z_{B0}\left[j-k_{B}\right]$$

It is worth mentioning that no restriction has been put on the definition of vectors ${\vec{Z}}_{A0}$ and ${\vec{Z}}_{B0}$, so that arbitrarily complex winding layouts can be used in the proposed model, including the uniform distribution of the conductors along the slot width, or along skewed slots.

### 4.2. Vector ${\vec{L}}_{AB}$ Containing the Mutual Inductances between Phases A and B for All Their Relative Positions

With the vectors defined in Section 4.1, the expression of the mutual inductance between phase *A*, with its axis located at an angular position $\varphi_{A}=k_{A}\cdot \Delta \varphi$, and phase *B*, placed at other angular position $\varphi_{B}=k_{B}\cdot \Delta \varphi$, $L_{k_{A}k_{B}}$, can be formulated, using Equation (22), as

(42) $$L_{k_{A}k_{B}}=\sum_{i=0}^{N-1}\sum_{j=0}^{N-1}Z_{Bk_{B}}\left[i\right]\cdot Lp_{i}\left[j\right]\cdot Z_{Ak_{A}}\left[j\right]$$

Equation (42) can be formulated with vectors ${\vec{L}}_{p0}$, ${\vec{Z}}_{A0}$, ${\vec{Z}}_{B0}$, using Equations (34), (39), and (41) respectively, as

(43) $$L_{k_{A}k_{B}}=\sum_{i=0}^{N-1}\sum_{j=0}^{N-1}Z_{B0}\left[i-k_{B}\right]\cdot Lp_{0}\left[j-i\right]\cdot Z_{A0}\left[j-k_{A}\right]$$

Using the change of variables $j^{\prime }=j-k_{A}$, Equation (43) becomes

(44) $$L_{k_{A}k_{B}}=\sum_{i=0}^{N-1}\sum_{j^{\prime }=0}^{N-1}Z_{B0}\left[i-k_{B}\right]\cdot Lp_{0}\left[j^{\prime }-\left(i-k_{A}\right)\right]\cdot Z_{A0}\left[j^{\prime }\right]$$

and, using the change of variables $i^{\prime }=i-k_{A}$, Equation (44) becomes

(45) $$L_{k_{A}k_{B}}=\sum_{i^{\prime }=0}^{N-1}\sum_{j^{\prime }=0}^{N-1}Z_{B0}\left[i^{\prime }-\left(k_{B}-k_{A}\right)\right]\cdot Lp_{0}\left[j^{\prime }-i^{\prime }\right]\cdot Z_{A0}\left[j^{\prime }\right]$$

That is, the mutual inductance between phases *A* and *B* depends only on their relative position $(k_{B}-k_{A})\cdot \Delta \varphi$. In this way, it can be defined a vector ${\vec{L}}_{AB}$ whose element *k*, $L_{AB}\left[k\right]$, contains the mutual inductance between phases *A* and *B* for an angular separation between them k·Δφ

(46) $$L_{AB}\left[k\right]=\sum_{i=0}^{N-1}\sum_{j=0}^{N-1}Z_{B0}\left[i-k\right]\cdot Lp_{0}\left[j-i\right]\cdot Z_{A0}\left[j\right]$$

Equation (46) is simple to setup, because it contains only the expression of the partial inductance between two axial conductors (Equation (32)), and the distribution of the conductors (not the coils) of phases *A* and *B*, all of them obtained using a common origin of coordinates. However, at the same time, it is cumbersome to calculate, because a double summation is needed for obtaining the mutual inductance $L_{AB}\left[k\right]$, for each value of *k* at *N* different positions. Nevertheless, this process can be performed in an extremely efficient way using the FFT, as proposed in the present work.

### 4.3. Matrix Formulation of the Expression of the Mutual Inductance between Phases A and B

Equation (45) can be expressed in matrix form as

(47) $${\vec{L}}_{AB}={{\mathrm{TcZ}}_{B0}}^{T}\cdot {\mathrm{TcL}}_{p0}\cdot {\vec{Z}}_{A0}$$

where ${\mathrm{TcL}}_{p0}$ is a square N×N matrix defined as

(48) $${\mathrm{TcL}}_{p0}=\left[\begin{array}{cccc} L_{p0}\left[0\right] & L_{p0}\left[N-1\right] & \cdots & L_{p0}\left[1\right]\\ L_{p0}\left[1\right] & L_{p0}\left[0\right] & \cdots & L_{p0}\left[2\right]\\ \vdots & \vdots & \ddots & \cdots \\ L_{p0}\left[N-1\right] & L_{p0}\left[N-2\right] & \cdots & L_{p0}\left[0\right] \end{array}\right]$$

and ${{\mathrm{TcZ}}_{B0}}^{T}$ is the transpose of a square N×N matrix defined as

(49) $${\mathrm{TcZ}}_{B0}=\left[\begin{array}{cccc} Z_{B0}\left[0\right] & Z_{B0}\left[N-1\right] & \cdots & Z_{B0}\left[1\right]\\ Z_{B0}\left[1\right] & Z_{B0}\left[0\right] & \cdots & Z_{B0}\left[2\right]\\ \vdots & \vdots & \ddots & \cdots \\ Z_{B0}\left[N-1\right] & Z_{B0}\left[N-2\right] & \cdots & Z_{B0}\left[0\right] \end{array}\right]$$

Matrix ${\mathrm{TcL}}_{p0}$ is built using the column vector ${\vec{L}}_{p0}$ in Equation (36) as its first column, and rotating each successive column by one position. That is, ${\mathrm{TcL}}_{p0}$ is a Toeplitz circulant matrix. Matrix ${\mathrm{TcZ}}_{B0}$ has been built using the same procedure, thus it is also a Toeplitz circulant matrix.

### 4.4. Computation of the Mutual Inductance between Phases A and B Using the FFT

A nice property of the Toeplitz circulant matrices is that they become diagonal by a transformation into the frequency domain. Therefore, Equation (48) can be reduced to a simple element-wise multiplication of three vectors in the frequency domain, using the FFT (and its inverse, the IFFT), as

(50) $${\vec{L}}_{AB}=\mathrm{IFFT}\left(\mathrm{FFT}{\left({\vec{Z}}_{B0}\right)}^{*}\;\circ \;\mathrm{FFT}\left({\vec{L}}_{p0}\right)\;\circ \;\mathrm{FFT}\left({\vec{Z}}_{A0}\right)\right)$$

where the superscript ${}^{*}$ stands for the conjugate of each vector element, and the operator ∘ stands for the element-wise or Hadamard product of two vectors (written as “*” in MatLab environment). This equation is similar to the one presented in [72], where the convolution theorem has been applied to obtain the mutual inductances between two phases, based on the magnetomotive force generated by a single conductor, instead of the MVP.

Equation (50) gives, with a single element-wise multiplication of three vectors in the frequency domain, the values of the mutual inductance between phases *A* and *B* for every angular displacement between them (with a precision Δφ). It relies only in the distribution of the phases’ conductors, and on the analytical expression of the partial inductance between two axial conductors, Equation (32), which makes it very simple to implement. This approach differs from the approaches followed in [70,71], where the equation of the MVP in cylindrical coordinates (Equation (26)) must be solved again for each distribution of phase conductors, which limits its practical application to simple distributions, such as the sinusoidal ones.

The derivative with respect to the angular coordinate of the mutual inductance between phases *A* and *B*, located on opposite surfaces, has the same expression as Equation (50), just replacing the sequence ${\vec{L}}_{p0}$ in Equation (32) by its angular derivative, the sequence ${\vec{dL}}_{p0}$ in Equation (35), giving

(51) $$\frac{\partial {\vec{L}}_{AB}}{\partial \varphi }={\vec{dL}}_{AB}=\mathrm{IFFT}\left(\mathrm{FFT}{\left({\vec{Z}}_{B0}\right)}^{*}\;\circ \;\mathrm{FFT}\left({\vec{dL}}_{p0}\right)\;\circ \;\mathrm{FFT}\left({\vec{Z}}_{A0}\right)\right)$$

**Figure 6:**
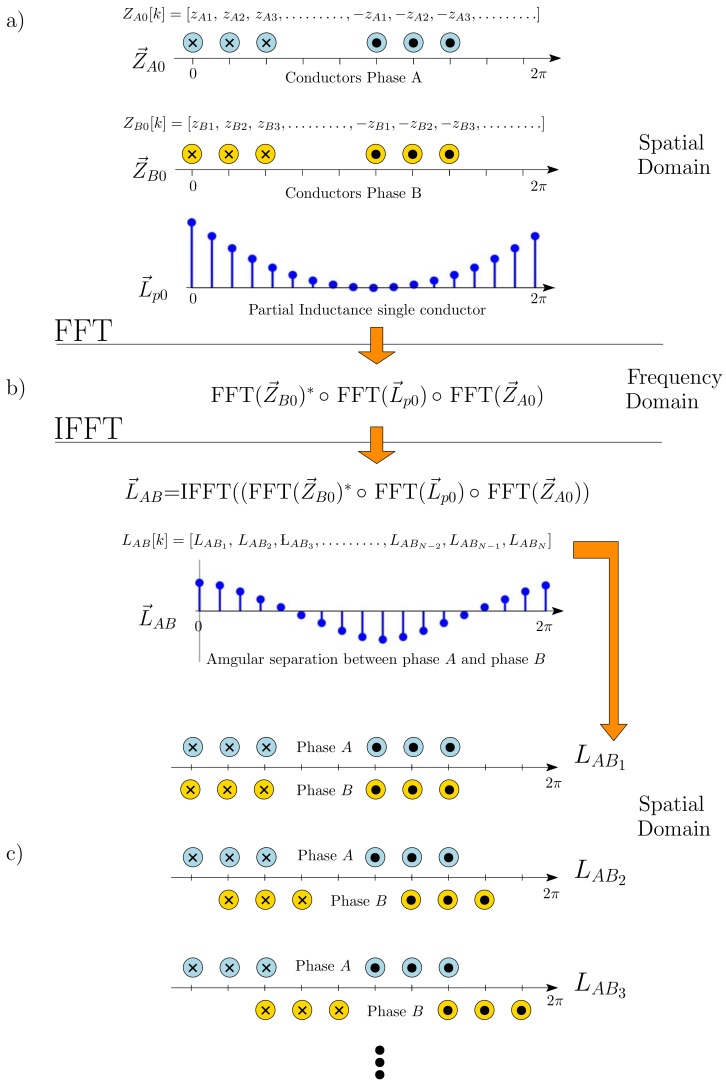
Block diagram for the computation of the mutual inductance between phases *A* and *B* with (50). (**a**) Construction of the vectors with the layout of the conductors of phases *A*, ${\vec{Z}}_{A}$, and *B*, ${\vec{Z}}_{B}$, and the partial inductance between a conductor placed at the origin and other conductor at successive positions, ${\vec{L}}_{p0}$; (**b**) Element-wise multiplication in the frequency domain of these three vectors; (**c**) Conversion into the spatial domain of the result of the multiplication, which gives the vector ${\vec{L}}_{AB}$. The element *k* of this vector, $L_{AB}\left[k\right]$, is the mutual inductance between phases *A* and *B* for a relative angular position between them of k×2π/N.

The procedure for obtaining the mutual inductance between two phases *A* and *B*, ${\vec{L}}_{AB}$ in Equation (50), has been depicted graphically in the block diagram shown in Figure 6.

The same block diagram in Figure 6 is valid for computing the derivative of the mutual inductance between phases *A* and *B*, ${\vec{dL}}_{AB}$ in Equation (51), just replacing in Figure 6 the vector ${\vec{L}}_{p0}$ in Equation (36) by its derivative, the vector ${\vec{dL}}_{p0}$ in Equation (37).

## 5. Experimental Validation

The proposed method has been applied to the fault diagnosis of a commercial squirrel-cage induction motor, whose characteristics are given in Appendix A. The type of fault that has been used for the experimental validation of the proposed model is a broken bars fault. The use of the proposed partial inductance approach is particularly well suited for modeling a squirrel cage induction motor with broken bars. In this case, instead of using rotor meshes formed by two consecutive bars, as usually done, the faulty rotor winding can be modeled using directly the bar currents in Equation (10), and the partial inductances of the rotor bars in Equations (7), (8) and (11). Following this approach, the presence of multiple broken bars, consecutive or not, can be introduced in the model just by eliminating the broken bars entries in the current and voltage vectors, Equations (9) and (10) , instead of modifying the inductances of the rotor meshes affected by the broken bars. This approach avoids the cumbersome process of recomputing the rotor inductances matrix to take into account the variations in some rotor meshes generated by the broken bars fault.

It is worth mentioning that the election of a commercial squirrel cage motor for performing the experimental validation of the proposed method does not exclude its validity for other types of induction machines, such as wound rotor induction machines. In fact, Equations (50) and (51) do not impose any constraint on the winding distribution, and are equally valid for both single rotor bars and wounded rotor phases.

### 5.1. Experimental Setup

**Figure 7:**
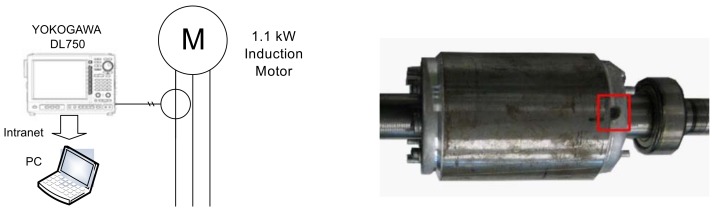
Experimental setup: measurement equipment (**left**); and rotor of the motor whose characteristics are given in Appendix A, with a broken bar (**right**).

The test equipment, displayed in Figure 7, consists of a current clamp, whose characteristics are given in Appendix B, a 200 pulse/revolution incremental encoder, a Yokogawa DL750 Oscilloscope and a Personal Computer connected to it via an intranet network. As shown in Figure 7, the broken bar fault has been artificially produced by drilling a hole in the selected bars.

**Figure 8:**
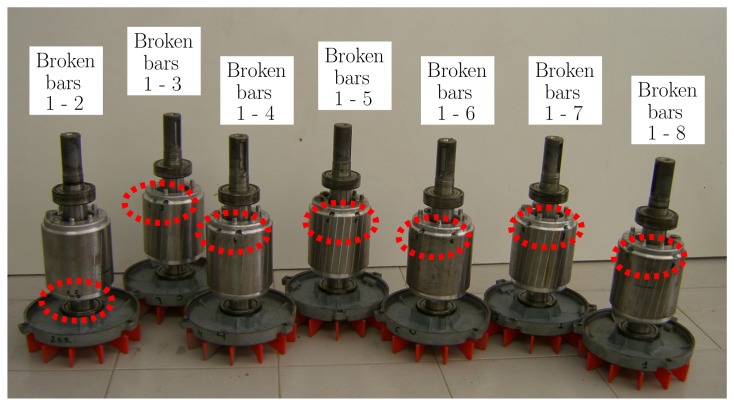
Tested rotors with two broken bars in different relative positions.

The experimental validation of the proposed approach has been carried out using a set of artificially damaged rotors with one or two broken bars, beginning with two consecutive broken bars (Positions 1–2), and increasing gradually their separation (Positions 1–3, 1–4, etc.), up to a separation of seven slots between the two broken bars (Positions 1–8), as shown in Figure 8. An additional healthy rotor, with no broken bars, has also been used for comparison purposes. This gives a total number of nine experimental tests: a healthy motor, a motor with one broken bar, and seven motors with two broken bars at different positions.

**Figure 9:**
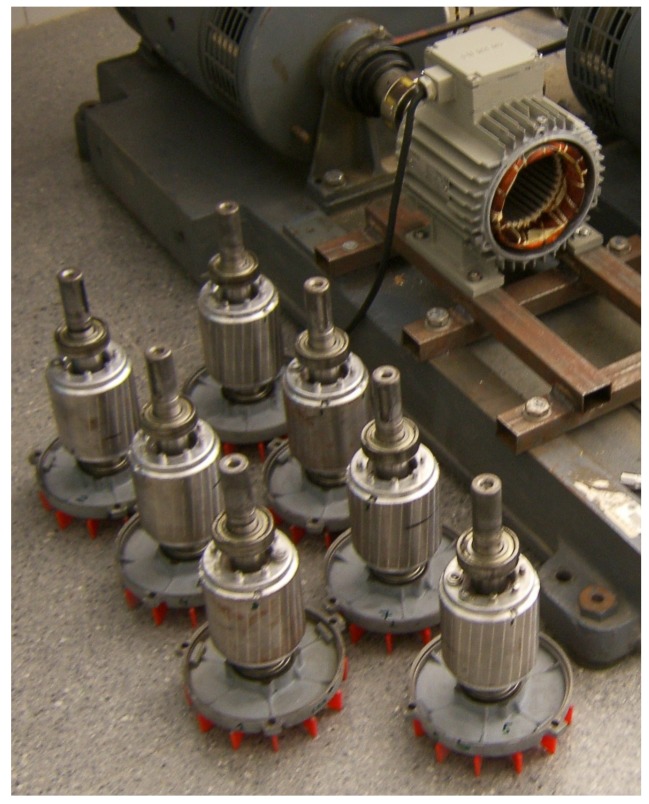
Tested rotors with two broken bars in different relative positions, with the stator used for performing all the experimental tests.

**Figure 10:**
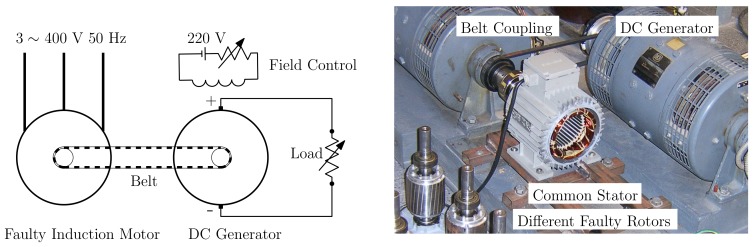
Schema of the loading of the experimental machine (**left**) and experimental setup (**right**). The induction motor under test (Appendix A) is connected to a DC generator via a belt coupling. The DC machine feeds a resistive load. Both the resistive load and the field excitation can be controlled so that the induction machine works at rated speed.

The same stator has been used in all the experimental tests (Figure 9), which allows comparing the effects of the broken bar fault in the stator currents in a controlled environment. To this end, the motors were disassembled, and one of the stators was mounted in the test bed. All tests have been carried out mounting the various rotors (see Figure 9) in this stator. The induction motor under test (Appendix A) is connected via a belt coupling to a DC generator, which feds a resistive load (see Figure 10). Both the resistive load and the field excitation of the generator can be controlled, so that the induction machine works at rated speed 1410 r/min (*s* = 0.06). The current of a stator phase has been measured using a sampling frequency of 5000 Hz, during an acquisition time of 50 s, and its spectrum has been obtained for identifying the fault harmonics given by Equation (1).

### 5.2. Model Setup

**Figure 11:**
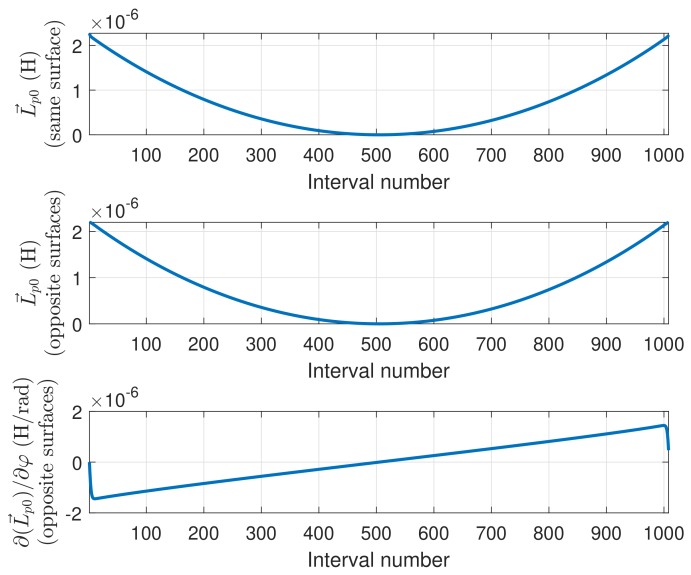
Partial inductance between two conductors of the simulated machine (32), if the conductors are in the same surface (**top**), or in opposite surfaces (**middle**). Derivative of the partial inductance with the angle (35), when the conductors are in opposite surfaces (**bottom**).

The partial inductance between two conductors of the simulated machine in Equation (32) has been represented in Figure 11, for the cases of the two conductors lying in the same surface (Figure 11, top), or in opposite surfaces (Figure 11, middle). The derivative of the mutual inductance of a stator conductor and a rotor one in Equation (35) has been also represented in Figure 11, bottom. These values depend only on the geometrical dimensions of the machine.

**Figure 12:**
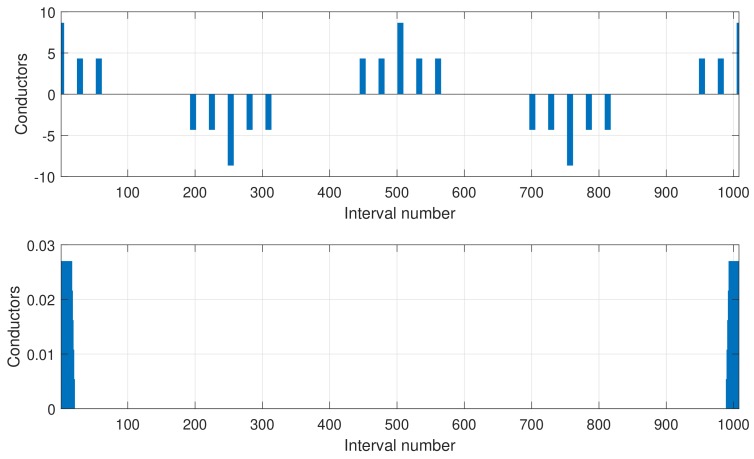
Distribution of the conductors of a stator phase (**top**) and a rotor bar (**bottom**).

The distribution of the conductors of a stator phase and the one that corresponds to a skewed rotor bar of the simulated machine are shown in Figure 12.

**Figure 13:**
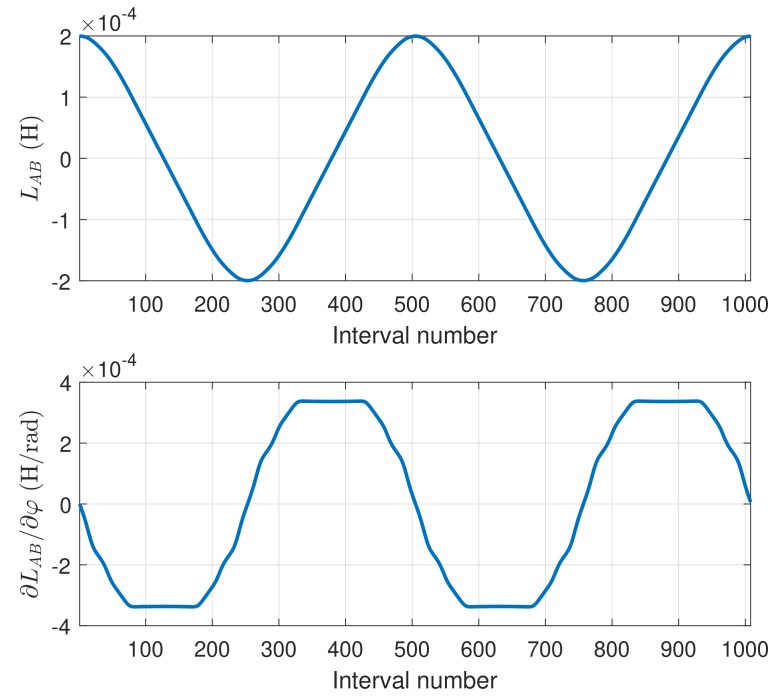
Mutual inductance between a stator phase and rotor bar of the simulated machine as a function of the rotor position (**top**), and its angular derivative (**bottom**).

A direct substitution in Equations (50) and (51) of the values presented in Figure 11 and Figure 12 gives the mutual inductance between two phases, for every relative angular position, and its derivative respect to the angular coordinate. The mutual inductance between a stator phase and a rotor bar of the simulated machine, as a function of the rotor position, is given in Figure 13, top, and its angular derivative is given in Figure 13, bottom.

The simulations with the motor model have been carried using a constant load torque $T_{L}$ in Equation (12), with a value equal to the motor rated torque, that is, $T_{L}=9550\times 1.1/1410=7.45$ Nm.

### 5.3. Diagnosis of a Single Broken Bar Fault

**Figure 14:**
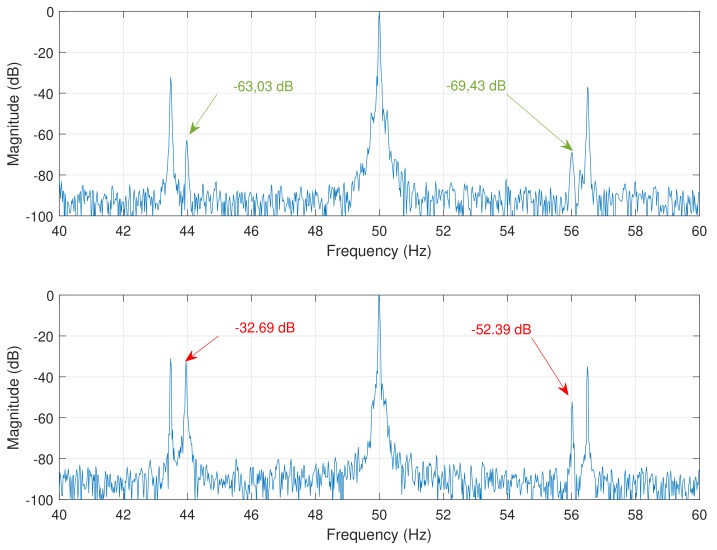
Spectrum of the experimental motor in healthy conditions (**top**); and with a single broken bar (**bottom**). The position and magnitude of the fault harmonics (LSH and USH) are indicated with arrows.

**Figure 15:**
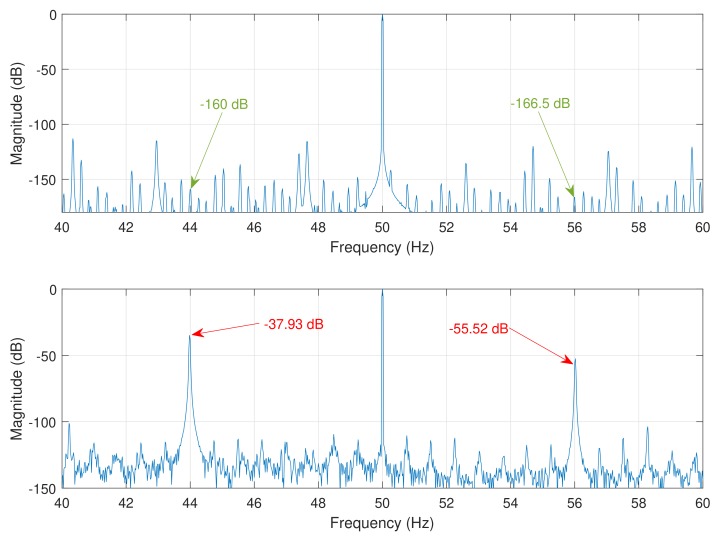
Spectrum of the simulated motor in healthy conditions (**top**); and with a single broken bar (**bottom**). The position and magnitude of the fault harmonics (LSH and USH) are indicated with arrows.

The procedure for the diagnosis of a single broken bar fault is based on the MCSA technique. In the case of a broken bar fault, the magnitude of the characteristic fault harmonics given by Equation (1) increases significantly. For the tested and simulated motors, the rotor speed is the rated one (1410 r/min , $f_{1}=50$ Hz). The main fault harmonics used for the diagnosis are those corresponding to a value k=±1 in Equation (1): the lower sideband harmonic (LSH), with a frequency $f_{LSH}=\left(1-2s\right)f_{1}$, and the upper sideband harmonic (USH), with a frequency $f_{USH}=\left(1+2s\right)f_{1}$. In the case of the tested and simulated motors, s=0.06, so that $f_{LSH}=\left(1-2\times 0.06\right)\times 50=44$ Hz, and $f_{USH}=\left(1+2\times 0.06\right)\times 50=56$ Hz. Figure 14 shows the spectrum of the currents measured in the experimental tests, with a healthy and a faulty motor with a single broken bar. Figure 15 shows the same spectra obtained from the simulated motor.

The magnitude of the fault harmonics are presented in Table 1, showing a good agreement between the simulated and the experimental data. It is worth mentioning that, in the case of the experimental motor, two additional harmonics appear at frequencies of 43.5 Hz and 56.5 Hz, due to the belt used for coupling the load to the test bed. In fact, when the motor is tested unloaded, with the belt removed, these harmonic do not appear, so they are probably generated by an axial eccentricity induced by the asymmetric load coupling to the motor shaft. These harmonics do not appear in the case of the simulated motor, because the partial inductances model presented in this work does not take into account the effect of axial eccentricity.

### 5.4. Diagnosis of a Double Breakage Fault with Non-Consecutive Broken Bars

**Figure 16:**
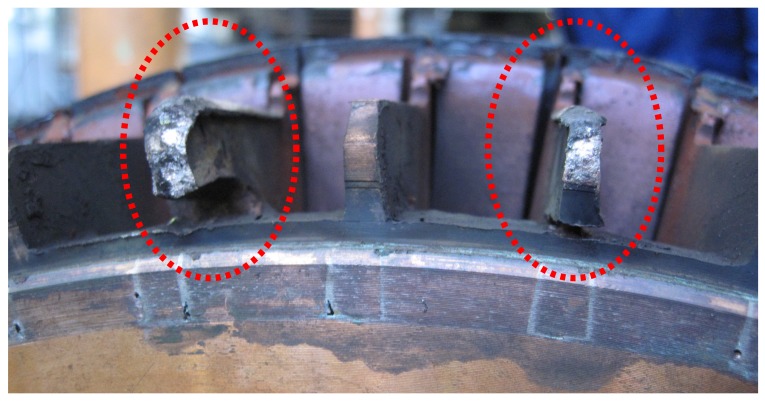
Rotor of an induction machine of 3.15 MW, 6 kV, with two non-consecutive broken bars.

Major motor manufacturers have reported cases where the damaged bars appear randomly distributed around the rotor perimeter, indicating that the failure of non-adjacent bars is fairly common in large cage induction motors. Figure 16 shows the rotor of a 3.15 MW, 6 kV induction motor with a double breakage fault, affecting to non-consecutive rotor bars.

In the case of a double breakage, the magnitude of the LSH is a function of the relative position of the two broken bars. In [73] it has been shown that the ratio between the LSH in the case of double and single breakages depends on the angle between the broken bars as Equation

(52) $$LSH_{pu}=|\frac{LSH_{double}}{LSH_{single}}|=\left|2\cos\left(p\alpha_{bb}\right)\right|$$

where *p* is the number of pole pairs and $\alpha_{bb}$ is the angle between the two broken bars. From Equation (52), it can be deduced that if $\alpha_{bb}$ approximates π/2p, that is, half a pole pitch, then the second breakage reduces the magnitude of the LSH to a value lower than its magnitude in the case of a single breakage. Therefore, in this case, a motor with two broken bars could be erroneously diagnosed as a healthy motor. This behavior is more challenging to simulate than the single broken bar fault, and it has been selected to verify the validity of the proposed model, based on partial inductances, for fault diagnosis.

**Figure 17:**
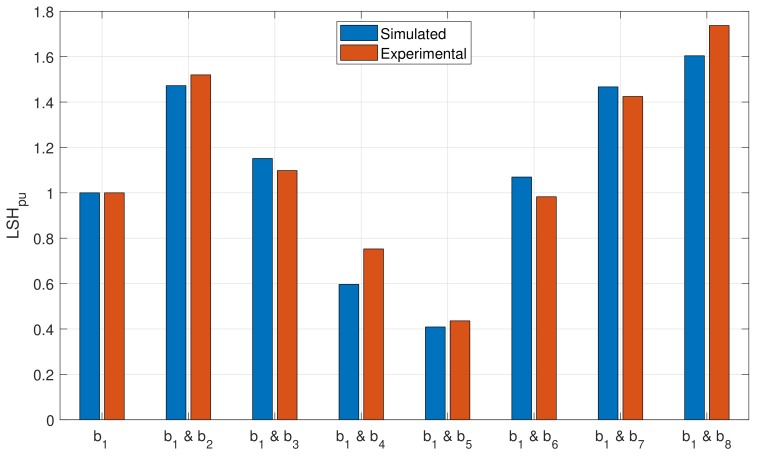
Comparison between the simulated and the experimental data obtained for the motor with one broken bar (first column), and with two broken bars separated a variable number of healthy bars.

The following procedure has been followed to perform the experimental tests:

1. Seven rotors in healthy condition have been successively mounted and tested, to verify that there are no significant differences between the tested rotors.
2. A bar has been drilled in each of the rotors, denoted as $b_{1}$ bar. The motors have been tested at rated speed, and, using the spectrum of one of the stator phase currents, the magnitude of the LSH ($LSH_{single}$ in Equation (52)) has been recorded.
3. A second bar has been drilled at different positions from the first one (see Figure 8). The motors with a double breakage have been tested again at rated speed, and, using the spectrum of one of the stator phase currents, the magnitude of the LSH ($LSH_{double}$ in Equation (52)) has been recorded.
4. Finally, the ratio $LSH_{pu}$ has been computed for every rotor, and compared with the same value obtained with the simulated motor. The results are presented in Figure 17 and in Table 2. The time spent in each simulation (50 s in steady state) was 12 s, using a computer whose characteristic are given in Appendix C.

The results shown in Figure 17 and in Table 2 indicate that the results obtained with the proposed model clearly follow the experimental trend, which confirms its validity as a tool for fault diagnosis. It is worth mentioning that, with the proposed approach, all the experimental tests, using the rotors presented in Figure 8, have been simulated using a single inductances matrix, which contains the partial inductances between stator phases and rotor bars. Each type of the considered faults has been simulated just by eliminating the faulty bars from the set of Equations (7), (8) and (11), without any modification to the rest of the terms in these equations.

## 6. Conclusions

The development, improvement and implementation of fault diagnostics techniques for induction machines requires the use of fast and accurate dynamic models of the machine, which can take into account the asymmetries generated by the machine faults. In this paper, a novel approach has been proposed, using the concept of partial inductance. Instead of using the coil as the basic winding unit, the partial inductance between conductors has been proposed for building the matrices of phase inductances and their derivatives. The partial inductance of a single conductor has been obtained analytically using the magnetic vector potential, and the combination of the partial inductances of all phases has been solved using the FFT, which makes the proposed approach very fast to compute and very easy to implement. The proposed method has been theoretically presented and experimentally validated using the diagnosis of double breakage faults in the squirrel cage of a commercial induction motor for different, non-consecutive positions of the broken bars.

The application of this novel approach is not limited to the specific fault that has been used to perform the experimental validation, namely a broken bars fault. Other types of fault that can be simulated with the procedure presented in this paper include rotor asymmetries of wound-rotor inductions machines, stator inter-turn faults, bearing faults, oscillating loads and supply imbalances. Future work will extend this approach to induction machines with non-uniform air gap, which will allow the simulation of eccentricity faults (static, dynamic and mixed), and the effect of core saturation. In these cases, the analytical computation of phase inductances is a highly complex issue. The proposed method can alleviate this complexity by computing analytically only the MVP generated by a single conductor, which, inserted in the formulae presented in this work, Equations (50) and (51), can provide seamlessly the phases mutual inductances, regardless the complexity of the windings layout.

## Figures and Tables

**Table 1:** Magnitude of the main fault harmonics generated by a broken bar fault in the tested and simulated motors.

	Healthy Motor		Faulty Motor	
	LSH	USH	LSH	USH
Experimental	−63.03 dB	−69.43 dB	−32.69 dB	−52.39 dB
Simulated	−160 dB	−166.5 dB	−37.93 dB	−55.52 dB

**Table 2:** Comparison between the ratio $LSH_{pu}$ of Equation (52) computed with the experimental tests and with the simulated motors.

	$LSH_{pu}$	
	Experimental	Simulated
Single broken bar ($b_{1}$)	1	1
Broken bars $b_{1}$ & $b_{2}$	1.52	1.472
Broken bars $b_{1}$ & $b_{3}$	1.098	1.151
Broken bars $b_{1}$ & $b_{4}$	0.7527	0.5965
Broken bars $b_{1}$ & $b_{5}$	0.4358	0.4089
Broken bars $b_{1}$ & $b_{6}$	0.9827	0 1.07
Broken bars $b_{1}$ & $b_{7}$	1.425	1.467
Broken bars $b_{1}$ & $b_{8}$	1.737	1.604

## Appendix A. Commercial IM

Three-phase induction machine. Rated characteristics: P=1.1kW, f=50Hz, U=230/400V, I=2.7/4.6A, n=1410r/min, cosφ=0.8.

Machine dimensions: Effective length of the magnetic core = 70.2 mm, radius at the middle of the air gap = 41.1 mm, air gap length = 1.2 mm.

Stator: Three-phase winding, 36 slots, 78 wires/slot, winding pitch = 7/9, slot opening width = 2.1 mm, phase resistance 7.68 Ω, end winding leakage = 2.3 mH.

Rotor: Squirrel-cage winding, 28 bars, slot opening width = 1.4 mm , skew = one slot pitch, bar resistance = 0.00202 mΩ, end winding leakage = $2.45\times 10^{-5}$ mH.

## Appendix B. Current Clamp

Chauvin Arnoux MN60, Nominal measuring scope: 100 mA–20A, ratio input/output: 1 A/100 mV, intrinsic error: ≤ 2% + 50 mV, frequency use: 400 Hz–10 kHz.

## Appendix C. Computer Features

CPU: Intel Core i7-2600K CPU @ 3.40 GHZ RAM memory: 16 GB, Matlab Version: 9.4.0.813654 (R2018a).
